# Supplementary material for: Investigating the origin and consequences of endogenous default options in repeated economic choices
Source: PLoS One. 2020 Aug 13;15(8):e0232385. doi: 10.1371/journal.pone.0232385 (PMC7425902; doi:10.1371/journal.pone.0232385)
Supplement: Attachment — Submitted filename: ResponsetoReviewers.docx [file pone.0232385.s002.docx]

**Reviewer #1:**

In the manuscript “Investigating the origin and consequences of endogenous default options in repeated economic choices”, Couto and colleagues aim to investigate the existence and properties of endogenous default options. Authors adopt a standard economic binary choice task with varying monetary amounts and probabilities of gains or losses. By probing the effects of three experimental features including time pressure, time spent on task and relative choice proportion on, authors dissociated between two features of endogenous default options: natural default (a natural tendency to prefer certain types of options) and learned default (the tendency to implicitly learn a default option from past choices). Moreover, authors show that the natural default option bias results from a distortion of the decision-making process under time pressure, while the learned default option bias results from the emergence of an independent process endorsing the most frequently chosen option with increasing time on task.

The manuscript is well written and describes an interesting question about endogenous default options in economic choices. I have a few suggestions and a major comment on the effect of ‘learned default option’ that should be addressed to strengthen the conclusions of the paper. Please find my comments below:

We thank the reviewer for his/her positive evaluation of our manuscript and his/her constructive comments that we have now addressed.

Major concerns:

- Page 9, line 213; Is there any reason why authors do not consider a probability weighting function in their fit?

We thank the reviewer for this suggestion. Probability weighting function (PWF) is an important addition of Prospect Theory over Expected Utility Theory, and accounts for decision-makers overweighting small probabilities and underweighting of large probabilities [1]. The PWF (subjective probability as a function of objective probability) hence typically has an inverse S-shape form. This implies probability distortions to be more extreme near the endpoints of the probability scale than near the middle. However, in the present study, all probabilities in the stimuli set (both P and 1-P) are middle range (20-80) – i.e., in the ‘linear’ region of the inverse S-shape form. Therefore, we do not expect that including PWF would dramatically improve and/or modify our Expected Utility fit.

Besides, note that the simple expected utility value function used appeared sufficient to design stimuli sets that achieved the desired choice patterns (as estimated in the priming sessions).

We, nonetheless, think the idea of investigating default effects on PWF quite interesting, with a slightly different experimental task. This is now discussed in Stimuli (lines 243-246) and General Discussion (lines 557-560).

Lines 243-246: *It should be noted that this model does not take into consideration probability distortions [41], as probabilities in our stimuli set (both P and 1-P) are middle range (20-80) – i.e. in the ‘linear’ region of the inverse S-shape form. Probability distortions would typically be observed on the extremes of the probability range, which we did not measure.*

Lines 557-560: *Although probability distortions are not considered in our model to generate lotteries (see Stimuli), it would be of interest for delineating and refining the actual properties of defaults options if future studies investigate the effects of such probability distortions* *with a slightly different stimuli set [59].*

- Related to PWF, a previous study on primates’ choice behavior [2], has shown a change in probability distortion function as a result of repeated choice sequence. How is the ‘learned default option’ different than a change in PWF that can happen as a result of over-exposure to a certain type of options? How does a change like that effect authors claims? Please comment on this.

We thank the reviewer for this question and the reference. The referred study [2] investigates the effect of different types of trial sequence structures on risky choices – via the shape of probability distortion. The authors report that the shape of probability distortion, the typical inverse S-shape, is reversed in certain conditions – i.e. when trial sequences are repeated. These experimental setup and results are of interest for delineating and refining the actual properties of defaults options.

Yet, and in line with our previous response, we want to draw the reviewer’s attention to the fact that probabilities in our stimuli set are middle range, which should clearly attenuate – or even negate – any effect of probability distortions – which are characteristics of extreme probabilities.

Also, please note that the referred study uses non-human primates. As is typical in such studies all trials and decisions have to be followed by outcomes (rewards). In humans, such experiments are called “decision-by-experience” and contrasts with “decisions-by-description” – where options are only described and not “reinforced”. Our current study, on the other hand, typically leverages decision-by-description experiments. Critically, the inversion – or at least modulation – of PWF between decisions by description vs experience is well documented and known as the *description-experience gap* [3,4]. Whether default options similar to the one we currently describe are present in non-human primates or other animals is an outstanding question, but experimentally investigating this question might face important difficulties. We know discuss this point in our General discussion (lines 597-602):

Lines 597-602: *Whether default options similar to the ones we describe in this study are present in non-human primates or other animals is an outstanding question. For example, it has been shown that non-human primates attitude towards risky choices can change with trial sequence repetition [59]. Yet, we are aware that experimental attempts to replicate or extend the present results in non-human animals might face important challenges.* *If, on one hand, in animal studies all decisions have to be followed by outcomes (i.e., rewards), on the other hand, the presence of feedback after choices has been repeatedly shown to elicit different decisions patterns in humans – a phenomenon called description-experience gap [74,75].*

Finally, please note that all lottery pairs in our study are unique – i.e., no repetition, neither in the form of intermixed nor separated blocks. The interpretation of the “learned default option” as a probability distortion reversal and as a result of over-exposure to a certain type of options is thus not straightforward either, at least, under the current experimental task/design. This has been clarified in lines 87-89.

Lines 87-89: *It should be noted that repetition of the most frequent choice type in both dominant and learned default options refers to the repetition of individuals’ own choices and not to stimuli repetition.*

Minor concerns:

- To increase readability, I suggest the authors to add their explanation of fixed-point property to the introduction section. This way, the introduction section includes all the required information to understand the authors’ claims and results in the abstract.

We agree that the Introduction should contain all the relevant information of the study. We now further explain the fixed-point property at the end of the Introduction (lines 103-109).

Lines 103-109: *We assessed the relative merits of these hypotheses by quantifying how RT distributions associated with the experimental manipulations mimic properties of binary mixtures under different mixture proportions, capitalizing on the so-called fixed-point property of binary mixture distributions [33,34]. The fixed-point property refers to the fact that different mixture proportions of the same two base distributions share a common probability density point. In the present study, this feature can be used as a statistical test of a quite conservative specification of dual-process hypothesis: if we observe a fixed-point for RT distributions observed under different experimental conditions, then RTs are likely to be generated by two independent processes, whose mixture proportion was modulated by the experimental manipulation.*

- I would recommend authors to use ‘experience’ or some other terminology to refer to the effect of experience with task. The phrase ‘time spent on task’ was a bit confusing to me. Without the explanation in the main text, I thought authors are referring to reaction time in choice trials where there in no time pressure.

The term “time on task” is quite a literal one, and quite common in Cognitive Psychology literature [5,6]. It refers to the time participants spend on a task, which is exactly what we want to refer to. Although we understand the suggestion, in our view, “experience” alludes more to the effects that the time spent on task factor has than to the factor itself. In addition, a detailed explanation is given when the default options hypotheses are elaborated. For these reasons, we think it is reasonable to keep the term time on task.

- I suggest that authors show ‘No Default’ case first instead of last in Fig. 1.

The no default case is now displayed first in figure 1. Caption is updated accordingly (lines 141 and 152).

Fig 1. Hypotheses about the underlying causes of default options.

- Please avoid using top/bottom to refer to figures. Authors should consider adding labels to Fig. 1 bottom row and Fig 4B.

The terms top/bottom panel are replaced, whenever possible, by A and B in the revised manuscript. That is the case of figures 3 and 5. Captions are updated accordingly.

- Please add a sentence explaining what asterisks represent in Fig. 2 and Fig. 5.’s captions.

The asterisks represent the significance level of time pressure and of interaction between choice proportion and time on task (i.e., the main results). A sentence to clarify this is added in figure 3 and 5.

**: P<0.05 for the main effect of time pressure and the interaction between choice proportion and time on task.*

- Page 17, line 418; It would be helpful to provide a figure or a statistical comparison of fixed-point analysis for Experiments 1 and 2, confirming the assumption that there is no difference between the two experiments.

As explained in the study, we aggregate data from both experiments (Experiment 1 and 2) when performing the fixed-point property analysis. The reason why we do it is closely related to the hypothesis on the endogenous default options mechanisms, which should be independent on the investigated frame (i.e., gain and loss frame) – i.e., the fixed-point property should yield similar results regardless of the frame. Considering this theoretical consideration and the potential statistical advantages – e.g., in terms of statistical power – , we do think it is reasonable to perform a common analysis. This is not the case for the hypotheses made on default options choice patterns and that is why, in that case, we do perform separate analysis. We, nonetheless, provide figures of the fixed-point property for Experiment 1 and 2 for illustration purposes (Fig R1-2). Note that the patterns of results are very consistent across the gain and loss frames, and the conclusions do not change – i.e., likely presence of a fixed-point for time on task (BF = 6, gain frame and BF=7, loss frame).

Fig R1. Fixed-point property, Experiment 1.

Fig R2. Fixed-point property, Experiment 2.

**Reviewer #2:**

This paper studied the existence and properties of endogenous default options in risky decision making. To do that, the authors manipulated and investigated the effects of three experimental features, time pressure, time spent on task and relative choice proportion towards a specific option. They found and dissociated two features of endogenous default options which could bias individuals’ choices, the natural default and the learned default. Moreover, the natural default biases the choice process towards an option category, while the learned default effects may be attribute to another process.

This study is interesting, but has the following problems:

We thank the reviewer for his/her insightful comments who helped us improve our manuscript.

Also, with respect to the reviewer’s negative response to “*3. Have the authors made all data underlying the findings in their manuscript fully available?*” we want to draw the reviewer’s attention to the fact that all data are fully available without restriction. This is specified in the data availability section of the submission. *All data are fully available in an OSF repository, DOI 10.17605/OSF.IO/TSJBU*.

(1) I am a little bit confused about the term “default”. Is it really the default option or a prior bias? How do you know it is the default option?

We agree with the reviewer that *default options* can be seen as *a priori biases*. Actually, we feel that those two conceptualizations could be used interchangeably. The main difference seems to be a disciplinary one: the term default option is typically used in behavioral economics (see e.g., [7] , while *a priori bias* are more used in e.g. perceptual decision making, due to the field strong influences from Bayesian Theory and Signal Detection Theories [8,9]. We briefly discuss this subtlety in the Introduction (lines 53-58).

Lines 53-58: *Endogenous default options are, thus, based on the same core idea as the exogenous default options (i.e., the tendency to stick with a default-option and, thereby, avoid alternative-options), with the substantial difference that in the exogenous case, the default options are explicitly framed to individuals (e.g., as “opting-in” or “opting-out”), not internally framed and built by individuals.* *In this respect, endogenous default options are very similar to a priori biases studied, for example, in the perceptual decision literature [11,12]*

(2) The authors related the default to the automatic or fast process in dual-process theories, is the automatic process the default process? This is also related to the question above. Is it a default bias, or an automatic bias, or a prior bias?

The automatic or fast process from dual-process theories is a very general umbrella term. Our study dissociates several potential dimensions of endogenous default options which may concurrently exist and be revealed/dissociated under specific experimental manipulations, and all of which could be – at first sight – linked to an automatic and fast process under dual-process theory assumptions. We understand that the dissociation of several dimensions may be a bit confusing, however, we do argue that dissociations like this are extremely important to overcome somewhat vague theoretical and experimental specifications that dual-process theories have been criticized for [10]. Especially if we consider that these dimensions, although potentially underpinned by different neuro-cognitive processes, could be confounded under simpler experimental designs, and lead to mis-interpretations and contradictory results [11]. This point is now explicitly addressed in the General Discussion (lines 537-538).

Lines 537-538: *The novel contribution of the current study, which follows from the careful control over multiple experimental factors, is the first dissociation of several potential dimensions of endogenous default options (natural, dominant and learned default options (…)), all of which could be – at first sight – linked to an automatic and fast process, a very general umbrella term under dual-process theory assumptions.*

(3) In the abstract, the authors mentioned that “the learned default effects may be attributable to a second independent choice process.” What is the “second independent choice process”? Could you please make it more explicitly?

The attribution of the learned default effects to a second independent choice process is made based on the stringent assumption of the fixed-point property, for which we found evidence. It assumes that two processes can execute the decision in a strictly independent manner. Following this rationale, we think it is reasonable to conclude that the learned default option bias results from the emergence of an explicit strategy or a heuristic-like process, for which individuals are expected to apply “simple rules” – here, in the form of simple repetition of the most frequent choice type – that they learn implicitly through experience, and through the course of the task. Importantly, such process is assumed to be qualitatively independent from the process in charge of the decision in the beginning of the task. We now explicitly address this interpretation in the General Discussion (lines 584-586).

Lines 584-586: *This suggests that the emergence of the learned default is due to the emergence of an independent decision-process, like an explicit strategy or a heuristic-like process [68], for which individuals apply ‘simple rules’ – here, in the form of simple repetition of the most frequent choice type – that they learn implicitly through experience, and through the course of the task.*

(4) The “dominant default” is induced by the mere repetition of the most frequent choice type, and the “learned default” is induced over time by the repetition of the most frequent choice type. In this sense, are these two default endogenous or exogenous?

Our study investigates *endogenous* default options. *Exogenous* default options imply that the default options are *explicitly framed to individuals* (e.g., as opting-in or opting-out). This differs substantially from the endogenous default options which imply individuals to internally frame decisions and build default options in the absence of such explicit exogenous cues, even if induced and/or modulated by specific experimental factors – i.e., time pressure, in the case of the natural default, time pressure * most frequently chosen option in the case of the dominant default, and time on task * most frequently chosen option in the case of the learned default.

In the case of the dominant and learned defaults, specifically, we should emphasize that the repetition of the most frequent choice type refers to the repetition of participants’ own choices and not to stimuli repetition per se – this is controlled in our study and is unique every trial. That said, we still think that these defaults can be categorized as endogenous – i.e., arising from an individual's internal state. We clarify this point now in the Introduction (lines 87-89).

Lines 87-89: *It should be noted that repetition of the most frequent choice type in both dominant and learned default options refers to the repetition of individuals’ own choices and not to stimuli repetition.*

(5) Figure 1 needs more explanations. It is not clear how the authors get the figure.

We apologize for the lack of clarity. We now address this more clearly in the Introduction (lines 117-119). Time pressure has typically been used to reveal endogenous default options – i.e., whether individuals are naturally inclined to be risk-averse vs risk-seeking. Despite its prominence, results have been inconsistent. In this study, we argue that such inconsistencies may be explained by some other concurrent factors that are often neglected and/or not analyzed in the field. We specifically consider the time spent on task, which correlates with the learning of task contingencies and efficient strategies, and the most frequently chosen option, which correlates with the lottery set composition and/or the average risk preferences observed in the sample of participants. Figure 1 illustrates our hypotheses about the effects of these factors, and operationalizes them in terms of default-options, which can be identified via their specific choice patterns.

Lines 117-119: *Fig 1 schematically illustrates how the hypothesized default options affect choice behavior under the following experimental manipulations: time pressure, time spent on a task, and relative choice proportion towards one specific option.* *In contrast to time pressure, which has been typically used in studies from behavioral economics to reveal endogenous default options [19,20], the two last factors have often been neglected and/or not analyzed in the field. Importantly, they could explain the inconsistencies between results of different studies.*

We acknowledge that other factors could also have an effect on choice behavior, however, because their choice patterns are not consistent with the results of a preliminary experiment, we do not consider them (see also Additional Behavioral Predictions – and Supplementary Figure 3 – in S1_File).

(6) “Top panels” and “Down panels” in Figure 3 should be “Left panels” and “Right panels”?

We thank the reviewer for spotting this error. The revised manuscript displays Figure 3A and 3B, respectively. Caption is updated accordingly.

(7) “The results show that participants predominantly chose the option designed to be the most frequently chosen; participants increasingly chose the risky option with increasing time pressure, regardless of the most frequently chosen option; and participants increasingly chose the most frequently chosen option while they progressed through the experiment… “All these results are at the aggregate/group level, is there any individual differences? For instance, most of them go to the same direction, but others go to the opposite direction.

Our study was designed as a specific combination of within and between-subject experimental manipulations, aiming at testing precise hypotheses – at the group level – about default-options. We did not have any specific a priori hypothesis on individual differences, nor did we design our study to investigate those safely (e.g., in terms of statistical power, or endogeneity issues – see e.g., [12]). We do acknowledge, however, that individual differences are an interesting topic of discussion. Therefore, we reproduced figures 3 and 5 with individual traces in transparency for illustration purposes here. Looking at Fig R3-4 we can see that, although the probabilities reported in the priming session are very close to the expected probabilities, there is a significant between-subject variability. Likewise, although the reported choice patterns are in line with the proposed natural and learned default options, there is also a significant between-subject variability in those. This may be a reflection of strategy differences between participants, which deserves further investigation, though in future studies, where this question of individual differences could be better investigated, with customized experimental and statistical designs.

Fig R3. Observed proportion of safe choices with individual traces, Experiment 1.

Fig R4. Observed proportion of safe choices with individual traces, Experiment 2.(8) More explanations are needed for the fixed-point property. Why the fixed-point property exists when the observed data are the result of a mixture of two base distributions, and why not for a single process?

We agree that the fixed-point property deserves more attention in the Introduction. We therefore added an additional explanation of the method and its application to hypothesized dual processes (lines 103-109).

Lines 103-109: *We assessed the relative merits of these hypotheses by quantifying how RT distributions associated with the experimental manipulations mimic properties of binary mixtures under different mixture proportions, capitalizing on the so-called fixed-point property of binary mixture distributions [33,34]. The fixed-point property refers to the fact that different mixture proportions of the same two base distributions share a common probability density point. In the present study, this feature can be used as a statistical test of a quite conservative specification of dual-process hypothesis: if we observe a fixed-point for RT distributions observed under different experimental conditions, then RTs are likely to be generated by two independent processes, whose mixture proportion was modulated by the experimental manipulation.*

Note that the full mathematical demonstration of this property, as well as the description of its implementation to test hypotheses in behavioral sciences and potential limitations are described in the two referred publications:

33.	Van Maanen L van, Jong R de, Rijn H van. How to Assess the Existence of Competing Strategies in Cognitive Tasks: A Primer on the Fixed-Point Property. PLOS ONE. 2014;9: e106113. doi:10.1371/journal.pone.0106113

34.	Van Maanen L, Couto J, Lebreton M. Three Boundary Conditions for Computing the Fixed-Point Property in Binary Mixture Data. PLOS ONE. 2016;11: e0167377. doi:10.1371/journal.pone.0167377

(9) In Figure 4A, the authors mentioned the red dot indicates the fixed-point, but there is no red dot in Figure 4A.

We thank the reviewer for spotting this omission. Figure 4A is correctly updated in the revised manuscript.

(10) Studies have used sequential sampling models to investigate the process underlying binary decisions and the biases in the decision process. Why the authors do not use sequential sampling models?

Again, note that our study was designed as a specific combination of within and between-subject experimental manipulations, aiming at testing precise hypotheses – at the group level – about default-options with simple tests (regressions). We did not design the study with the aim to explore mechanistic decision models with sequential sampling models (SSMs). We find that the statistical test that we use to test the hypotheses on the endogenous default options mechanisms: the fixed-point property is more informative to the debate about default-options and/or dual processes of cognition.

We should emphasize, however, that the fixed-point property and sequential sampling models (as potentially other models) can be complementary and inform each other. This point is now discussed explicitly in the General Discussion (lines 586-590).

Lines 586-590: *Future studies investigating the effects of these default options using model-based approaches (e.g., sequential sampling models), would be a good complement to the fixed-point property analysis. Here, we focused on the fixed-point property, because it specifically tests hypotheses about single versus dual processes – an outstanding question with respect to the link between default options and dual-process models of cognition.*

**References**

1.	Gonzalez R, Wu G. On the Shape of the Probability Weighting Function. Cognit Psychol. 1999;38: 129–166. doi:10.1006/cogp.1998.0710

2.	Ferrari-Toniolo S, Bujold PM, Schultz W. Probability Distortion Depends on Choice Sequence in Rhesus Monkeys. J Neurosci. 2019;39: 2915–2929. doi:10.1523/JNEUROSCI.1454-18.2018

3.	Hau R, Pleskac TJ, Kiefer J, Hertwig R. The description–experience gap in risky choice: the role of sample size and experienced probabilities. J Behav Decis Mak. 2008;21: 493–518. doi:10.1002/bdm.598

4.	Heilbronner SR, Hayden BY. The description-experience gap in risky choice in nonhuman primates. Psychon Bull Rev. 2016;23: 593–600. doi:10.3758/s13423-015-0924-2

5.	Coull JT, Frackowiak RSJ, Frith CD. Monitoring for target objects: activation of right frontal and parietal cortices with increasing time on task. Neuropsychologia. 1998;36: 1325–1334. doi:10.1016/S0028-3932(98)00035-9

6.	Olofsson JK, Polich J. Affective visual event-related potentials: Arousal, repetition, and time-on-task. Biol Psychol. 2007;75: 101–108. doi:10.1016/j.biopsycho.2006.12.006

7.	Jachimowicz J, Duncan S, Weber E, Johnson E. When and why defaults influence decisions: a meta-analysis of default effects. Behav Public Policy. 2019; 1–28. doi:10.1017/bpp.2018.43

8.	Ratcliff R, McKoon G. The Diffusion Decision Model: Theory and Data for Two-Choice Decision Tasks. Neural Comput. 2008;20: 873–922. doi:10.1162/neco.2008.12-06-420

9.	Mulder MJ, Wagenmakers E-J, Ratcliff R, Boekel W, Forstmann BU. Bias in the brain: a diffusion model analysis of prior probability and potential payoff. J Neurosci Off J Soc Neurosci. 2012;32: 2335–2343. doi:10.1523/JNEUROSCI.4156-11.2012

10.	Melnikoff DE, Bargh JA. The Mythical Number Two. Trends Cogn Sci. 2018;22: 280–293. doi:10.1016/j.tics.2018.02.001

11.	Krajbich I, Bartling B, Hare T, Fehr E. Rethinking fast and slow based on a critique of reaction-time reverse inference. Nat Commun. 2015;6: 7455. doi:10.1038/ncomms8455

12.	Duncan GJ, Magnuson KA, Ludwig J. The Endogeneity Problem in Developmental Studies. Res Hum Dev. 2004;1: 59–80. doi:10.1080/15427609.2004.9683330
